# Recommendations for the design of small population clinical trials

**DOI:** 10.1186/s13023-018-0931-2

**Published:** 2018-11-06

**Authors:** Simon Day, Anneliene Hechtelt Jonker, Lilian Pek Lian Lau, Ralf-Dieter Hilgers, Ilan Irony, Kristina Larsson, Kit CB Roes, Nigel Stallard

**Affiliations:** 1 Clinical Trials Consulting & Training Limited, 53 Portway, North Marston, Buckingham, Buckinghamshire MK18 3PL UK; 2 IRDiRC Scientific Secretariat, Inserm US-14, Paris, France; 3 Department of Medical Statistics, RWTH Aachen University, Aachen, Germany; 4 Center for Biologics Evaluation and Research/ Office of Tissues and Advanced Therapies, US Food and Drug Administration, Silver Spring, USA; 5 Orphan Medicines, European Medicines Agency, London, UK; 6 Julius Center for Health Sciences and Primary Care, UMC Utrecht, Utrecht, The Netherlands; 7 Statistics and Epidemiology, Warwick Medical School, University of Warwick, Coventry, UK

**Keywords:** Small populations, Small population studies, Clinical trials, Statistical methods, Rare diseases, Recommendations, Patient engagement, IRDiRC

## Abstract

**Background:**

Orphan drug development faces numerous challenges, including low disease prevalence, patient population heterogeneity, and strong presence of paediatric patient populations. Consequently, clinical trials for orphan drugs are often smaller than those of non-orphan drugs, and they require the development of efficient trial designs relevant to small populations to gain the most information from the available data. The International Rare Diseases Research Consortium (IRDiRC) is aimed at promoting international collaboration and advance rare diseases research worldwide, and has as one of its goals to contribute to 1000 new therapies for rare diseases. IRDiRC set up a Small Population Clinical Trials (SPCT) Task Force in order to address the shortcomings of our understanding in carrying out clinical trials in rare diseases.

**Results:**

The IRDiRC SPCT Task Force met in March 2016 to discuss challenges faced in the design of small studies for rare diseases and present their recommendations, structured around six topics: different study methods/designs and their relation to different characteristics of medical conditions, adequate safety data, multi-arm trial designs, decision analytic approaches and rational approaches to adjusting levels of evidence, extrapolation, and patients’ engagement in study design.

**Conclusions:**

Recommendations have been issued based on discussions of the Small Population Clinical Trials Task Force that aim to contribute towards successful therapy development and clinical use. While randomised clinical trials are still considered the gold standard, it is recommended to systematically take into consideration alternative trial design options when studying treatments for a rare disease. Combining different sources of safety data is important to give a fuller picture of a therapy’s safety profile. Multi-arm trials should be considered an opportunity for rare diseases therapy development, and funders are encouraged to support such trial design via international networks. Patient engagement is critical in trial design and therapy development, a process which sponsors are encouraged to incorporate when conducting trials and clinical studies. Input from multiple regulatory agencies is recommended early and throughout clinical development. Regulators are often supportive of new clinical trial designs, provided they are well thought through and justified, and they also welcome discussions and questions on this topic. Parallel advice for multiregional development programs should also be considered.

## Background

The development and evaluation of drugs, biologics and devices, intended for the treatment of rare diseases, is often accomplished in small clinical trials. Rare diseases are characterized by a low prevalence. Different jurisdictions use different criteria for designating “rare” (or “orphan”): for example, fewer than 200,000 people affected by the condition in the US, fewer than 1 in 2000 people in the European Union, and fewer than 50,000 people in Japan [1–3]. However, many rare diseases may only affect a few hundred or a few thousand patients worldwide. Consequently, trials set up in these low prevalence conditions face many challenges, including heterogeneity in the patient population, difficulty in clinical trial recruitment, poorly understood natural history of the disease and often presentation in children, which brings additional challenges in clinical trials and trial design. To enhance the development of drugs for rare diseases, incentives have been implemented in the United States, the European Union and Japan, most notably through the US Orphan Drug Act of 1983 [1], the EU Regulation on orphan medicinal products (Regulation EC No 141/2000) [2], and in Japan Article 77–2 of the Pharmaceutical Affairs Law for orphan drugs [3] respectively. These incentives and others have shown moderate success [4–6] but the needs of patients with rare diseases are still insufficiently met at present [7]. This confirms the continuing need for efficient trial designs relevant to small populations to assure continued development of new treatments for rare diseases, especially when traditional randomised control trial designs are not possible due to limited number of available patients.

Further progress is needed in trial design aiming to assess medicines in a cost-effective, rigorously controlled manner, and in parallel with relevant analysis methods to assess treatment effects in small heterogeneous populations. These design options must also respect the need for reliable evidence prior to offering treatments to patients in need [8]. Therefore, developing design and analysis methodologies of clinical trials in small population settings requires a rigorous approach from all stakeholders, including regulators and patients. Several international initiatives and international actors are currently working to contribute to innovative solutions for small population studies [9–13].

### The IRDiRC Small Population Clinical Trials Task Force

The International Rare Diseases Research Consortium (IRDiRC) was set up in 2011 to coordinate and accelerate research, and to maximise scarce resources in the field of rare diseases [14]. It unites governmental and non-profit funding bodies, companies, umbrella patient advocacy organizations, and scientific researchers to promote international collaboration and advance rare diseases research worldwide. The Consortium is aimed at promoting international collaboration in rare diseases research and development worldwide, and has as one of its goals to contribute to the development 1000 new therapies for rare diseases. Its vision is to: Enable all people living with a rare disease to receive an accurate diagnosis, care, and available therapy within 1 year of coming to medical attention [15].

Three Scientific Committees (Diagnostics, Interdisciplinary, Therapies) advise the IRDiRC Consortium Assembly on research priorities and scientific progress. The Therapies Scientific Committee has issued recommendations [16] to address bottlenecks associated to health research in low prevalence but high need conditions, which are expected to foster the development of rare disease therapies on a global scale. Following issuance of these recommendations, IRDiRC launched several Task Forces to address specific issues. One of these is the Task Force on Small Population Clinical Trials (SPCT), set up to address the shortcomings of our understanding in small population clinical trials for rare diseases and to issue recommendations to ensure that methods used for such trials are conducive to ultimately making effective therapies available for patients [17]. Task Force members include researchers, industry representatives, regulatory agency representatives, and patient advocacy representatives. The Task Force held a workshop in March 2016 and this paper is based on the workshop outcome of this Task Force.

### Regulatory agency guidelines

Drugs for the treatment of rare diseases must demonstrate substantial evidence of clinical benefit in adequate, well-controlled studies. Different incentives exist for products that are granted orphan drug designation; however, no markedly different assessment standards are routinely used for orphan drugs in comparison to non-orphan drugs. The US Food and Drug Administration (FDA) and European Medicines Agency (EMA) have developed guidelines to support the design of clinical trials for small populations.

The EMA’s Committee for Medicinal Products for Human Use (CHMP) guideline states that “*no methods exist that are relevant to small studies that are not also applicable to large studies*” [18]. Nonetheless, the guideline also states that less commonly seen methodological designs may be acceptable in small population conditions if they might help improve the interpretability of the results of the study. The EMA highlights that the trade-off between small quantities of high quality evidence (from small randomised trials) and large quantities of lower quality evidence (from larger uncontrolled case series) must be considered and judged on a case-by-case basis. Marketing authorisation applications for orphan products tested in small populations are assessed according to the same standards as those for other products but consider limitations due to low patient recruitment. If randomised controlled trials are not possible, regulators are open to discuss the adoption of alternative methodologies and evidence sources to enhance the overall evidence base.

The FDA acknowledges the challenges of small population clinical trial development for rare diseases and has provided draft guidance to assist sponsors of drugs and biological products intended to treat or prevent rare diseases [19] wherein strategies were proposed for use in randomised control trial settings to support safety and efficacy claims in different drug development phases. In 2010, a draft “Guidance for Industry – Adaptive Design Clinical Trials or Drugs and Biologics” was published to provide guidance to sponsors for the development of adaptive clinical trials [20]. While being cautious to the risks associated with adaptive clinical trials, the document does provide guidance on modifications that can be planned in a prospectively written protocol, including eligibility criteria, randomisation procedure, total sample size, and endpoints. Caution should however be taken concerning the generalisability and applicability of adaptive clinical trial results. Various strategies may aim to decrease heterogeneity, improve disease characterization and predicted clinical course, which could thereby shape patient selection prior to randomisation but do not generally reduce statistical validity regarding the study population. The FDA furthermore encourages early communication to assist in drug evaluation, scientific, and medical questions that may arise throughout the clinical investigation. Better communication at clinical stages and around Special Protocol Assessments with the review divisions increases the chance of successful clinical outcomes.

The Pharmaceuticals and Medical Devices Agency (PMDA) in Japan has a guidance document for staff involved in new drug evaluations, entitled “Points to be considered by the review staff in the evaluation process of new drug” [21]. It is stated that - in particular for orphan drugs - final decisions should not be exclusively based on points covered in the document, but that points such as the clinical relevance should also be taken into account. Also, scientific evaluation should be based on the entirety of the document. No specific guidance document on small population studies is currently available but a guidance document on methodological issues related to these studies is expected to be developed. Nevertheless, PMDA offers consultation to give guidance and advice on clinical trials and on data for regulatory submissions. Specifically, for the development of orphan drugs with small population clinical trials, PMDA offers advice on alternatives to traditional methodologies at face-to-face meetings during early development stages.

### Paper outline

This paper outlines the recommendations of the IRDiRC SPCT Task Force that are structured around seven topics: “General recommendations”, “Different study methods/designs related to characteristics of medical conditions”, “Adequate safety data”, “Multi-arm designs”, “Decision analytic approaches and rational approaches to adjusting levels of evidence”, “Extrapolation”, and “Patients’ engagement in study design.” Each topic briefly explores the different recommendations set out by the Task Force to encourage, support and develop small population studies. The paper includes the results of the discussion concerning recent options and points to consider when designing small population clinical trials with special focus on rare diseases. The paper is not intended to give detailed explanations of methodological approaches – instead the reader should refer to common statistical texts or, in many cases, current research literature. A number of points for further discussion are also suggested, which future IRDiRC Task Forces (or other initiatives or regulatory agencies) might contribute to.

### Recommendations by the IRDiRC Small Population Clinical Trials Task Force

#### General recommendations

- When feasible, make full use of longitudinal data: Analysis methods for repeated measurements lead to a potential reduction of sample size by 30% versus change score analysis [22]. Such designs allow for the modelling of the development of the treatment effect, not just its existence (or otherwise) at a specified follow-up point. The question answered by such an analysis (or the “estimand”) is different to a simple analysis at a fixed time point, so the relevance of the question of “how the treatment effect develops” rather than “what is the effect at a given time” needs to be considered.
- Do not dichotomise continuous endpoints in the primary analysis (although possibly do so for sensitivity analyses and assessment of clinical relevance). Many measurements in medicine are inherently continuous measurements (for example, blood pressure) but there is often a wish to dichotomise patients as “responders” or “non-responders”. It is argued that differences in responder rates may have more clinical relevance than differences of means. Whether that is true or not may be case-dependent, but dichotomising will nearly always be an inefficient use of data, requiring more patients to demonstrate the existence of treatment effects.
- Trials should be long enough for complete follow up and patients should stay in trials for as long as possible to ensure adequate assessment of long-terms outcomes: in survival trials this can substantially reduce censoring, and in longitudinal data studies it increases the amount of information available. Unfortunately, additional costs can be connected to longer clinical trials but those costs should be balanced against the greater information gained from longer follow up.
- Use analysis of covariance (ANCOVA) instead of simple “change from baseline” analyses for reduction of bias and gains in efficiency [23]. ANCOVA is always more efficient than the similar “change from baseline” analysis. It accounts for imbalances at baseline more properly than “change from baseline” analyses, and it accounts for the correlation between baseline and endpoint measurements more properly than does a “change from baseline” analysis. ANCOVA need not be restricted to situations where the same variable is measured at baseline as at the trial endpoint.
- Use multiple endpoints, aimed at multiple study objectives, but with careful consideration of multiplicity problems [24]. Multiplicity problems are just as applicable in studies of rare diseases but the need to gain as much information from a limited patient population (in a rare disease) may be greater than that need in a more common condition.
- Use composites endpoints, by combining several outcomes into a single outcome measure, thereby increasing the number of events and hence the statistical power if treatment is likely to impact all single outcomes of the composite in the same direction. Composite endpoints are not without limitations and it is important to ensure all individual components of the composite are of similar clinical importance.
- Use different formulations, doses and endpoints if appropriate in different subpopulations and consider the possibility to combined analyses of these different groups. Examples of this may be when treating a wide age range when a treatment may have a similar effect in each age group, but the scales to measure the endpoint may need to be different. Methods to combine significance levels are well established [25], although less so for estimating the size of treatment effects.
- There is an ongoing need for rigorously collected natural history and patient registry data for rare diseases for the design of clinical trials. Natural history and patient registry data will both add to our understanding of how diseases progress and develop over time, but also assist in the determination of the endpoints where a clinically meaningful difference can be determined [26]. The FDA has recently awarded six research grants in this area, specifically to “inform medical product development by better understanding how specific rare diseases progress over time [27].” Given such understanding, it may become more viable to use natural history or patient registry data as an external control arm in clinical trials, rather than always relying on randomised controls.

### Different study methodologies related to characteristics of medical conditions

The gold standard for clinical trials is well known: randomised clinical trials with endpoints that are clear and meaningful to patients [28]. Nevertheless, adequately powered studies in rare diseases are not always a possibility. Trial designers should consider different design options and discuss their applicability with respect to efficiency and risk of bias. The applicability of each will depend, amongst other things, on the type of condition being studied – e.g. stable or highly variable, short or long-term endpoints, life limiting or symptom control, etc. Furthermore, risks of and opportunities for alternative trial designs should be discussed. Within the orphan drug development context, particularly in small trials, a discussion of appropriateness of the randomisation procedure as a design option is important. To assist choosing a suitable trial design, the following points should be considered:

- Whenever feasible, the gold standard - randomised clinical trial, with a clinically-relevant endpoint and long follow-up - should be used.
- For stable diseases with relatively short treatment duration, and where there are sufficient data to determine an appropriate washout period, cross-over designs [29] should be considered since these may allow potential large reductions in sample size. Such designs allow each subject to receive both (or perhaps more than two) interventions but will not be applicable in, for example, highly variable conditions or those where very long follow-up is needed. They are more applicable to relatively short term endpoints for stable diseases.
- Group-sequential designs [30]: The possibility of early stopping provides a potential reduction of sample size but they may also increase study size in some circumstances. Such designs incorporate interim analyses (which will usually necessitate use of a Data Monitoring Committee [31, 32]) and whilst they have advantages of potentially identifying early efficacy signals, they also may have the disadvantage of limited efficacy data in important subgroups and reduced the extent of safety data.
- Inferentially seamless adaptive designs [33, 34]: These designs can combine data from an exploratory “phase 2 part” of a trial (possibly for selecting appropriate doses) with data from the confirmatory “phase 3 part” of the *same* trial. There are many challenges with such designs, both in the time needed to design them and appropriate analyses to adjust for possible bias in estimates of treatment effect and proper control of type I error.
- If historical data are available, e.g. from registries or other control events, this may be helpful, but need to be properly weighted so that they do not overly influence a final trial conclusion more than the data from the trial [35, 36]. The relevance of historical data (considering changing diagnostic ability and changing standards of care) need to be carefully considered.
- Explore the options for trial designs that allow subjects to be used more than once (for example multiple n-of-1 trials, crossover trial designs or randomised withdrawal designs [37]). In particular, n-of-1 trials are an ideal tool for comparing the effects of multiple treatments in individual patients, but may be less suited to establishing treatment policies. They have many similarities with patient-preference designs [38].

It is recommended that trialists should always consider different design options, quantify what could be gained from each trial design, and carry out a comprehensive risk assessment before choosing a particular trial design. Many designs will often be possible, but the risks and benefits of each should be carefully evaluated to arrive at an evidence-driven choice of design. However, as well as creativity in trial design, it should be kept in mind that simplicity is often a virtue and unnecessary complexity of trial designs is to be discouraged.

### Adequate safety data

Safety is an essential component of the benefit-risk profile in the design of small population clinical trials. It is, however, never possible to state a definition of what constitutes adequate safety data as this depends on the nature of the disease and the product under investigation. In addition to the limited size of the intended population, the adequacy of the safety database depends on multiple factors, including the nature and severity of adverse events associated with the product during clinical development, the magnitude of benefit associated with the product in the studies that provides the primary evidence of effectiveness, and the patients’ tolerance for risk.

In small studies, clinical trial data alone typically do not give sufficient safety data. Therefore, it is important that several data sources are combined to give a fuller picture of the safety profile. Sources of data - not only restricted to clinical trial data - with a safety element in them include:

- Registry data
- Electronic health records (especially for drugs that are already in use, and that are considered as repurposing candidates)
- Non-clinical data (e.g. animal models). Such data almost always contribute to, for example, reproductive toxicity assessments but equivalent-dose (animal species-to-human) should also be considered when trying to predict on- and off-target toxicities.
- Post-marketing/ post-approval safety data (encourage health-care professionals to report adverse events)
- Extrapolation of data with similar compounds (questions should be considered about what would be the regulatory acceptability of these data; may be of interest for drug repurposing in specific populations)
- Data arising from comprehensive Risk Management Plans [39] agreed during, and initiated after licensing. Risk Management Plans are even more important in the setting of drug approvals being based on limited data since there are inevitably more “unknowns” than for a new product entering the marketplace based on fuller pre-approval data. Such a plan to continue to monitor a new medicine, and collect further data, should monitor aspects such as a medicine’s safety profile, how its risks will be prevented or minimised in patients, plans for studies and other activities to gain more knowledge about the safety and efficacy of the medicine, and measuring the effectiveness of risk-minimisation measures.
- Social media data (although ownership issues need to be addressed prior to usage)
- Use of product outside the clinical trial, e.g. in compassionate use setting that is “off label” but still covered by appropriate regulatory oversight [40].

Some of the above-mentioned sources are currently underused. It is recommended that researchers be better informed about the value of these data and how they might be used which, in return, will also improve contributions towards these different data sources. Additionally, to make better use of these sources, it is recommended that a prospective quality control system be put in place.

Regulatory approval should not put an end to collection of safety data; certainty and confidence will increase as post-marketing data are collected and analysed. There is, however, a balance needed between more data, potentially of lesser quality, and better controlled data. Improving the quality of the post-approval data should be given more attention and prioritised.

### Multi-arm designs

Multi-arm trials, platform trials, basket trials, etc. [41] are trials that incorporate several treatments in several treatment arms, each being tested for similar (although not necessarily identical) indications, possibly sharing a common control. The different treatments and trial arms might or might not start at the same time, and treatment arms might be added or dropped as the trial progresses. This trial design may be used in a proof-of-concept Phase II or, possibly, in a definitive Phase III trial. An example of multi-arm trial is the Phase II series of I-SPY trials [42, 43].

This topic was discussed separately from the general one of different study methodologies related to characteristics of medical conditions because many of those considerations can apply within a multi-arm design so that both sections need to be considered in parallel. Ideas and approaches within “platform trials” are more specific than, for example, a 3-arm trial of a new active vs. existing active vs. placebo.

Potential advantages of these types of trial designs are:

- Efficiency of potentially sharing the same control group
- Diminished chance for patients to receive a placebo, thereby encouraging participation
- Comparison of active substances
- Pooling data from active treatments, when feasible
- Sharing of resources, thus diminished trial costs, therefore contributing to efficient use of trial logistics.

Challenges of these trial designs are:

- In case trial treatments are from different sponsors, there is the need for sponsors to cooperate and agree to a common protocol
- Need for additional time to design such a complex study
- Challenges of operating the clinical trial
- Challenges of trial leadership
- Differences of interest between competitive companies, charities, investigators
- Potential heterogeneity and thus loss in efficiency due to heterogeneous settings.

Multi-arm trials should be considered as an opportunity by trial funders, patient organisations and other stakeholders for studies in rare diseases. Expertise centres, such as the European Reference Network for rare diseases, should try to channel patient flow towards this trial design if possible [44, 45]. Funders should be encouraged to fund multi-arm trials via international networks to test and compare multiple treatments more efficiently.

### Decision analytic and rational approaches to adjusting levels of evidence

If knowledge is available about a treatment, how do trialists make the best decisions, and which standards of evidence are required for decision making? In the topic of decision analytic and rational approaches to adjusting the level of evidence, three main questions were discussed.

The first, in addressing decision making when sufficient information is available for a treatment, several aspects should be considered, such as:

- Benefit-risk assessment – whilst *some* evidence for efficacy may exist, and concern about harms (adverse reactions to new medicines) will always exist, still the balance of benefits and risks may be acceptable. And the perspective of “acceptable” may differ for different stakeholders (see below).
- The patient horizon – i.e. in an (ultra) rare condition, the number of patients in the trial may be relatively “large” compared to the number who may potentially benefit (within a reasonable time frame) after the treatment becomes available. This may influence the decision concerning when there is enough evidence to start treating all eligible patients. Ethical judgment and the balance of benefits of faster access for patients to medicines compared with the benefits of attaining stronger evidence.
- The different stakeholder perspectives – however compassionate clinical trialists may be, the perspective of the patients will often be different to that of the researcher, the regulator, the prescriber, the purchaser, etc. In some cases, it may be just as appropriate to consider the public health impact of *not* giving treatment, as well as the more commonly considered risk (in terms of benefits vs. risks) of giving a treatment.
- The rate of innovation and how soon further (possibly better) alternative treatments may be expected to come to the marketplace.

The second question relates to the standards of evidence required in decision making. It is important to realise that the same standard of evidence may not be appropriate in every disease, especially when the number of patients who may benefit from the treatment is small. From a statistical perspective, some think significance levels less stringent than 5% (e.g. considering a *p*-value of 10% or 15% as indicative of a significant effect) should be accepted, while others believe that more stringent levels (e.g. *p*-values of 1%, or less) should be used. This indicates the potential to make decisions based on weaker levels of evidence compared to those of more common diseases. Additionally, although not relevant to regulatory approval, standards of evidence required by reimbursement bodies should also be taken into account.

The final question concerns technical issues regarding decision analytic approaches. These approaches extend beyond the relatively simply issue of analysing data and summarising what those data may say about benefits and harms, to the far more difficult problem of deciding what action to take (e.g. license a drug, or not; prescribe it, or not; pay for it, or not; take the medicine, or not, etc.). Such decisions inevitability rely partly on prior beliefs about the effects of a new therapy: one that is “very likely” to work may need less compelling data (from a trial) to convince someone to take it. In contrast, a new therapy that, for whatever reasons, seems “very unlikely” to be safe and effective will need more evidence to persuade someone to take it. Here, the term “prior information” comes into play and one issue often discussed is that of difficulties and methods for elicitation of informative Bayesian prior distributions [46].

No solid conclusions or recommendations were reached on this topic at the time the workshop was held, although it may be that such thinking in the context of rare diseases might trigger a fundamental re-think of the levels of evidence we typically require in many settings. In addition, the consortia of scientists formed by IDeAl [47], ASTERIX [48] and InSPiRe [49] have worked on these problems [10] (as well as many others).

### Extrapolation

Extrapolation is defined, according to the EMA, as “*extending information and conclusions available from studies in one or more subgroups of the patient population (source population), or in related conditions or with related medicinal products, to make inferences for another subgroup of the population (target population), or condition or product, thus reducing the need to generate additional information (types of studies, design modifications, number of patients required) to reach conclusions for the target population, or condition or medicinal product*” [50].

Extrapolation has been mostly discussed as a strategy to use for paediatric patient populations to address the practical and ethical difficulties they present when considering conducting clinical trials [51]. Extrapolation has been used to maximise the use of available data and to minimise the exposure of children to unnecessary clinical trials. On 1 April 2016, the EMA released a reflection paper on extrapolation [52]. Accordingly, data to support extrapolation of efficacy can come from many sources, not only pharmacokinetic/ pharmacodynamic models but also registries, off-label data, and electronic health records. The quantity and quality of data to be used for extrapolation, as well as the time for extrapolation (early phase trials, late phase trials), still need to be considered on a case-by-case basis. In the guidance paper from the FDA, it is described that if data already exist that are relevant to another (paediatric) indication and the evidence is of a scientifically rigorous nature, it may be possible to extrapolate the data in support of demonstrating a reasonable assurance of effectiveness, probable benefit and/or safety, but this approach should be used with care [53]. For sponsors that consider the use of extrapolation, it is recommended that they engage with regulators early in development planning.

There is currently limited experience in extrapolating from one rare disease study to another, or from one population to another, e.g. how to extrapolate data in the context of paediatric investigation plans. It is recommended that examples, preferably with clear illustration of their advantages, pitfalls and proper use be outlined and/or developed, including their methods of validation. There is also a gap in education which should be addressed; a common language between clinicians, biostatisticians, and modelling and simulation experts is needed.

### Patients’ engagement in study design

Patients’ voices and patient centeredness are essential in the set-up of clinical trials [35] but at present, there is no clear process describing how best to incorporate patients’ contributions in design and interpretation of trials. Patient involvement concerns many aspects of trial design including safety, benefit-risk assessment and endpoints. Consultation with patients experienced in clinical trials is advised, and the earliest consultation is preferred. When patient preference is important in regulatory decision-making, carefully designed patient preference studies should be considered as part of the clinical development process.

The process of incorporating patients’ feedback into the trial process is still relatively new to the pharmaceutical industry, therefore guidance - on the topics of when patient engagement is essential, how and when patient engagement should be sought, and from which particular group of patients - from regulators as well as patient organisations is highly valuable and should be provided.

The drafting of a best practice guidance document for the interactions between stakeholders of a clinical trial (sponsors, regulators, patients and patient advocates) is recommended. This best practice document should provide information about patient representation in trial design, focus on the potential benefit of consultation of patients and/or patient representatives, and how to manage potential conflicts of interest.

## Conclusions

This article described a summary of the expert discussions on methods for small population clinical trials that resulted from the IRDiRC SPCT Task Force workshop for rare diseases. These recommendations aim to contribute towards successful therapy development which ultimately leads to effective therapies becoming available to patients, and raise awareness of many possibilities for studies in rare diseases that all stakeholders should be aware of. While the randomised clinical trial is still considered the golden standard, it is recommended that trialists look systematically at alternative design options when setting up a clinical trial for a rare disease. Not every rare disease trial is as challenging as others, but if a randomised control design is not feasible, other trial options should be considered.

Combining all possible sources of safety data, i.e. longitudinal data, multiple endpoints, adjusting for baseline in the best possible way, is important to give a fuller picture of a therapy’s safety profile. Multi-arm trials should be considered an opportunity for rare diseases therapy development, and funders are encouraged to support such trial designs via international networks. Patient engagement is critical in trial design and therapy development, a process which sponsors are encouraged to incorporate when designing and conducting trials and clinical studies.

Better use of scientific advice from regulators regarding small population clinical trials should be promoted. Regulators are often supportive of new clinical trial designs, provided they are well thought through and justified, and they also welcome discussions and questions on this topic. Parallel advice for multiregional development programs should also be considered.

## Abbreviations

ANCOVA: analysis of covariance
CHMP: Committee for Medicinal Products for Human Use
EMA: European Medicines Agency
FDA: US Food and Drug Administration
IRDiRC: International Rare Diseases Research Consortium
PMDA: Pharmaceutical and Medical Devices Agency
SPCT: Small Population Clinical Trials
